# Feces and liver tissue metabonomics studies on the regulatory effect of aspirin eugenol eater in hyperlipidemic rats

**DOI:** 10.1186/s12944-017-0633-0

**Published:** 2017-12-11

**Authors:** Ning Ma, Xiwang Liu, Xiaojun Kong, Shihong Li, Zenghua Jiao, Zhe Qin, Pengcheng Dong, Yajun Yang, Jianyong Li

**Affiliations:** Key Lab of New Animal Drug Project of Gansu Province; Key Lab of Veterinary Pharmaceutical Development, Ministry of Agriculture, Lanzhou Institute of Husbandry and Pharmaceutical Science of Chinese Academy of Agricultural Sciences, No.335, Jiangouyan, Qilihe district, Lanzhou, 730050 People’s Republic of China

**Keywords:** Hyperlipidemia, Aspirin eugenol ester, Metabonomic, Biomarker, UPLC-Q-TOF/MS

## Abstract

**Background:**

Based on the pro-drug principle, aspirin and eugenol were esterified to synthesize aspirin eugenol ester (AEE). The anti-hyperlipidemia effect of aspirin eugenol ester has been confirmed in hyperlipidemic rat induced by high fat diet (HFD). However, its effect on liver and feces metabonomic profiles remains unknown.

**Methods:**

Suspension of AEE was prepared in 5% carboxymethyl cellulose sodium (CMC-Na). Thirty rats were divided into control, model and AEE groups. The control and model rats were fed with normal diet or HFD for 13 weeks, respectively. Rats in AEE-treated group were fed with HFD for 8 weeks to induce hyperlipidemia, and then given AEE once daily by oral gavage for 5 weeks at the dosage of 54 mg/kg body weight. After drug intervention, lipid profile analysis and oil red O staining were carried out to confirm the lipid accumulation in liver tissue. UPLC-Q-TOF/MS-based liver and feces metabonomics coupled with pathway analysis were conducted to evaluate the changes of metabolic profile and endogenous metabolites.

**Results:**

In liver tissue, oral administration of AEE significantly reduced lipid droplets and the levels of triglyceride (TG) and low-density lipoprotein (LDL). Using principal component analysis (PCA) and partial least squares-discriminate analysis (PLS-DA), distinct changes in metabolite patterns in feces and liver were observed. Liver and feces samples in control, model and AEE groups were scattered in PLS-DA score plots. 28 metabolites in liver and 22 in feces were identified as potential biomarkers related to hyperlipidemia. As possible drug targets, the perturbations of those biomarkers can be regulated by administration of AEE.

**Conclusion:**

Anti-hyperlipidemia effect of AEE was confirmed by lipid analysis, oil red O staining and metabolomics analysis. The mechanism of AEE might be associated with the changes in the metabolism of glycerophospholipid, amino acid, fatty acid, sphingolipid, purine, bile acid and glutathione.

**Electronic supplementary material:**

The online version of this article (10.1186/s12944-017-0633-0) contains supplementary material, which is available to authorized users.

## Background

Every year, about 15 million people die from cardiovascular disease (CVD) [1]. Related evidences demonstrate that hyperlipidemia is a crucial biomarker of CVD risk and has a close positive relationship with CVD. Hyperlipidemia is a metabolic disorder disease with abnormal levels of blood lipids such as the increasing of triglycerides (TG), total cholesterol (TCH), low-density lipoprotein (LDL) and the decreasing of high-density lipoprotein (HDL). It is found that the treatment of hyperlipidemia has a huge potential to reduce the mortality of CVD. Unfortunately, anti-hyperlipidemia drugs (such as statin and fibrate, etc.) have a number of adverse effects including liver injure, dementia risk, low plasma vitamin D levels and sleep disturbance [2]. Therefore, it is imperative to find more efficacious agents for the treatment of hyperlipidemia.

Aspirin, also known as acetylsalicylic acid, is a medication used to treat pain, fever, and inflammation. Moreover, increasing reports have demonstrated that aspirin has therapeutic effects on hyperlipidemia [3]. Eugenol is a natural product and safe essential oil, which is extracted from dry alabastrum of *Eugenia caryophyllata* Thumb. Evidences have proved that eugenol can significantly lower the levels of TCH, TG, and LDL in both serum and hepatic tissue samples from the rats with atherogenic diet [4]. However, the side effects of aspirin and eugenol limit their applications. For example, gastrointestinal damage is one of the constraint in clinical application of aspirin, which are mainly caused by the acidic group and direct contact of drug with gastric mucosa; Eugenol is structural instable and vulnerable to oxidation because of the free phenolic hydroxyl group. Based on the pro-drug principle, aspirin and eugenol are combined to synthesize aspirin eugenol ester (AEE) by esterification reaction [5]. AEE is a white and odorless crystal, which reduce the side effects of its precursors through the disappearance of free hydroxyl and carboxyl groups. Metabolism studies have proved that AEE can be decomposed into salicylic acid and eugenol after administration, and these metabolites can show their original activities and act synergistically [6]. The acute toxicity, subchronic toxicity and teratogenicity of AEE had been evaluated in our previous studies, which indicated that AEE was non-genotoxic in vitro or in vivo and its toxicity was lower than its precursors [7, 8]. These results suggested that AEE was a promising compound with good druggability.

Metabolomics, analysis of a huge range of small molecules in a biological system, is a sensitive and novel technology of systems biology in studies of pharmaceutical industry, disease diagnosis and nutrition. The metabolic profiling can provide a global view of abundance changes in endogenous metabolites in monitoring host responses to perturbations. Owing to high resolution, increased analytic speed, excellent sensitivity and powerful separation, UPLC–Q-TOF/MS has been proved to be a good technique for metabonomic analysis. Previous studies have demonstrated the advantage of LC-MS in the metabonomic studies related to hyperlipidemia, where they have revealed that the metabolic perturbations of energy, lipid, amino acids, bile acids, etc. were involved in the progress of hyperlipidemia [9].

Regulation effects of AEE on blood lipids in hyperlipidemic rats has been confirmed in our previous study, in which 54 mg/kg AEE significantly reduced TG, TCH, LDL, and elevated HDL [10]. Our previous plasma and urine metabonomic study has revealed that the effects of AEE against hyperlipidemia may involve in regulating the perturbation of glycerophospholipid metabolism, fatty acid metabolism, fatty acid beta-oxidation, amino acid metabolism, TCA cycle, sphingolipid metabolism, pyrimidine metabolism and gut microflora [11]. Meanwhile, feces and liver tissue, as important biological samples, may be attractive for biomarker investigation to provide a new insight into the progression of hyperlipidemia and the therapeutic basis of AEE. In this study, we applied fecal and liver tissue metabonomic strategies based on UPLC-Q-TOF/MS to illustrate the underlying mechanism of AEE against hyperlipidemia. Moreover, the lipid profile in rat hepatic tissue was also assessed.

## Methods

### Reagents and materials

Carboxymethylcellulose sodium (CMC-Na) was purchased from Tianjin Chemical Reagent Company (Tianjin, China). MS-grade formic acid was supplied by TCI (Shanghai, China). Deionized water (18 MΩ) was prepared with a Direct-Q®3 system (Millipore, USA). MS-grade acetonitrile was purchased from Thermo Fisher Scientific (USA). Oil red O was obtained from Sigma-Aldrich (St. Louis, MO, USA). The TG, TCH, LDL and HDL kits for serum were provided by Ningbo Medical System Biotechnology Co., Ltd. (Ningbo, China). The kits for measuring TG, TCH, LDL and free fatty acids (FFAs) in rat hepatic tissue samples were purchased from Nanjing Jiancheng Bioengineering Institute (Nanjing, China). Standard compressed rat feed and high diet feed (HFD) were supplied by Keao Xieli Feed Co., Ltd. (Beijing, China). Standard rat diet consisted of 12.3% lipids, 63.3% carbohydrates, and 24.4% proteins (kcal) and HFD (77.8% standard diet, 10% yolk power, 10% lard, 2% cholesterol and 0.2% bile salts) consisted of 41.5% lipids, 40.2% carbohydrates, and 18.3% proteins (kcal). AEE (transparent crystal, purity: 99.5% with RP-HPLC) was prepared in Key Lab of New Animal Drug Project of Gansu Province, Key Lab of Veterinary Pharmaceutical Development of Agricultural Ministry, Lanzhou Institute of Husbandry and Pharmaceutical Sciences of Chinese Academy of Agricultural Science.

### Animal treatments

Male Sprague-Dawley rats, aged 6 weeks and weighing 165–180 g, were purchased from Gansu University of Chinese Medicine (Lanzhou, China). Rat feed and drinking water were supplied ad libitum. Light/dark regimen was 12/12 h and living temperature was 22 ± 2 °C with relative humidity of 55 ± 10%. Animals were allowed a 2-week quarantine and acclimation period prior to start of the study.

### Drug preparation and experimental design

**Fig. 1:**
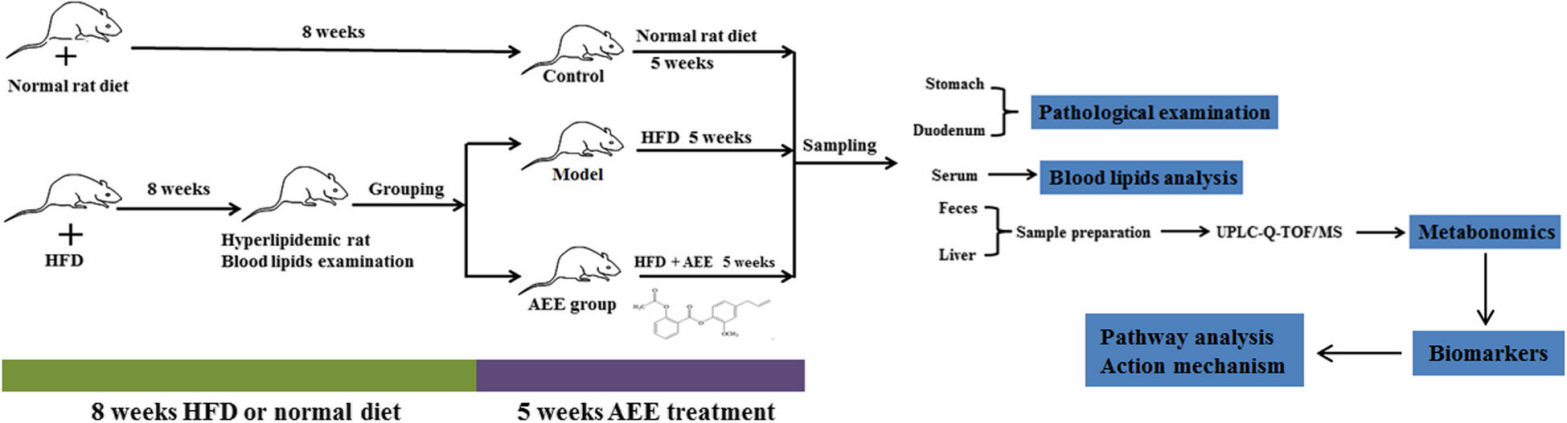
Schematic overview of the experiment design

AEE was ground and its suspension was prepared in 0.5% CMC-Na. The experimental design was shown in Fig. 1. Briefly, at the beginning of the experiments, rats were randomly separated into two groups. Group Ι as control group received standard diet (*n* = 10). Group II as model group received HFD (*n* = 20). The blood lipid levels were examined after HFD administered for 8 weeks (Additional file 1), and the results indicated that the hyperlipidemia disease was established successfully. After that, Group II was divided into two groups including model and AEE groups. Based on individual weekly body weight, AEE was intragastrically administrated at the dosage of 54 mg/kg. The rats in control and model groups were received equal suspension volume of 0.5% CMC-Na. The administration time of AEE was five weeks and HFD was continuously fed during the experiment period.

### Sample collection

Rats were fasted for 10–12 h before blood sampling. At the end of 8th week, rats were euthanatized with 10% chloral hydrate and then the blood samples (1–1.5 mL) were withdrawn from the tip of the tail into vacuum tubes to obtain serum samples (4000×*g*, 4 °C for 10 min). The serum samples were used for blood lipids analysis to evaluate the success of the hyperlipidemia model. At the end of experiment (13th week), all rats were euthanatized and sacrificed. Blood samples were taken from heart to prepare serum. Serum samples were frozen at −80 °C before biochemical analysis. Individual rats were placed in metabolic cages (1 per cage) to obtain 24-h fecal collections and fecal samples were stored at −80 °C before analysis. Stomach, duodenum and one part of liver tissue were fixed in 4% formalin for observations of pathological changes, and the remaining liver tissue was frozen at −80 °C until UPLC Q-TOF/MS analysis.

### Lipid profile and pathological examination

Serum samples were used to measure the levels of HDL, TCH, TG and LDL by using XL-640 automatic analyzer (Erba, Germany). Levels of TG, TCH, LDL and FFAs in rat hepatic tissue were also determined by assay kits. Under the standard protocol, tissues of liver, stomach and duodenum were formalin-fixed and paraffin embedded, sectioned, and stained with hematoxylin and eosin (HE). HE-stained sections were examined using a 13395H2X microscope (Leica, Germany). To further confirm lipid droplets accumulation, seven-micro frozen liver sections were stained by routine oil red O methods and lightly counterstained in Mayer’s hematoxylin solution.

### Sample preparation

The stool samples were lyophilized and then pulverized. Referring to previous study, weighted fecal samples were mixed with methanol at a ratio of 3 mL/g [12]. The mixtures were vigorously swirled for 60 s and treated with ultrasonic extraction, and then centrifuged at 10,000 rpm for 10 min at 4 °C. The supernatants were filtered through 0.22 μm nylon filter. An aliquot of 2 μL was injected for analysis.

Before analysis, 200 mg block of liver tissue was weighted precisely and mixed with 2 mL of chilled methanol/water (4:1, v:v), swirled for 1 min and homogenated to extract the compounds from liver [13]. Then, the homogenate was further ultrasonically broken for 8 min. After the samples were put in ice bath for 10 min, they were deproteinized by centrifugation at 4 °C (15,000 rpm, 10 min). Finally, 1.6 mL of the supernatant was evaporated with a vacuum dryer and reconstructed with 200 μL methanol/water (4:1, v:v) for UPLC-Q-TOF/MS analysis. An aliquot of 4 μL was injected for analysis.

### Data acquisition

Chromatographic separation of fecal and liver tissue samples were performed on an Agilent ZORBAX SB-C18 column (2.1 × 150 mm, 1.8 μm) using UPLC system consisted of a degasser, thermostat,two binary pumps and autosampler (1290, Agilent Technologies, USA). The column was maintained at 40 °C and eluted at a flowing rate of 0.3 mL/min, using a mobile phase of solvent A - water with 0.1% formic acid (by volume) and solvent B - acetonitrile with 0.1% formic acid (by volume). The optimized gradient programs for fecal and liver tissue samples were shown in Additional file 2.

Agilent 6530 Q-TOF (Agilent Technologies, USA) was used to carry out the mass spectrometry of plasma and urine samples with the electrospray ionization operating in positive (ESI+) and negative (ESI-) ion modes. The fragment voltage was set at 135 V in both modes. The capillary voltages were set at 4.0 KV in positive mode and 3.5 KV in negative mode, respectively. Used drying gas nitrogen, the desolvation gas rate was set to 10 L/min at 350 °C. The scan time was set at 1 spectra/s. Data was collected in centroid mode from 50 to 1000 *m/z*. The nebulizer pressure was set at 45 psig.

### Multivariate analysis and identification of potential biomarkers

Data processing method previously reported with minor modifications was used in this study [14]. The raw MS spectra were firstly processed by Mass Hunter Qualitative Analysis software (Agilent technologies, USA) to converted to common data format (.mzData). The program XCMS was used for nonlinear alignment of the data in the time domain and automatic integration and extraction of the peak intensities. The data were filtered by interquantile range and normalized for further multivariate data analysis. The obtained data sets were separately imported into SIMCA-P V13.0 (Umetrics AB, Sweden) to perform unsupervised principal component analysis (PCA) and supervised partial least squares discriminant analysis (PLS-DA). The quality of PLS-DA models was described by R^2^X, R^2^Y, and Q^2^. R^2^X and R^2^Y represented the fraction of the sum of squares for the selected component. Q^2^ represents the predictive ability of the model. To validate the model, permutation test was implemented (with 200 permutations). The candidate metabolites, with variance importance for projection (VIP) value above 1.0 and *P* value of ANOVA below 0.05, were considered to be potential biomarkers. Pathway analysis was performed with MetaboAnalyst, which is a web-based tool for visualization of metabonomics.

Identification of the metabolites was achieved through a mass-based search followed by manual verification. TOF-MS accurate mass value of the molecular ion of interest was searched against available biochemical databases such as the METLIN and Human Metabolome Database (HMDB). Then, MS/MS analysis was carry out to confirm the structure of potential biomarkers by matching the masses of the fragments. The pathway analysis of potential biomarkers was performed with MetaboAnalyst based on the pathway library of *Rattus norvegicus* (rat) to identify the metabolic pathways.

### Statistical analysis

The results of the blood lipids were expressed as mean ± standard deviation (SD). The significance of differences between Group I and Group II had been analyzed by Student’s *t*-test. The differences among three experimental groups had been evaluated by one-way ANOVA with Fisher’s least significant difference (LSD) test using the Statistical Package for Social Science program (SPSS 16.0, Chicago, IL, USA). The significance threshold was set at *P* < 0.05 for the test.

## Results

### Blood lipids analysis

The results of serum lipids were shown in Additional file 3. TCH, TG and LDL levels were significantly higher in the model group than that in the control group (*P* < 0.01), whereas the HDL was significantly reduced (*P* < 0.01). AEE showed strong effects on reducing TG, TCH and LDL than those in the model group (*P* < 0.01). With respect to HDL index, no statistical difference was observed between model and AEE treated groups. In comparison with the control group, HDL levels of AEE groups were significantly lower than control group (*P* < 0.01). TG, LDL and TCH levels in AEE group did not differ significantly from those in the control group, indicating the improvement of blood lipid levels in hyperlipidemic rats.

The levels of lipid profile parameters in hepatic tissue samples were shown in Table 1. When compared with the control group, HDF-fed rats showed significantly higher levels of TG, LDL and FFAs (*P* < 0.01). In AEE group, hepatic tissue levels of TG and LDL were significantly lower than those in the model group, while still higher than those in the control rats. The results showed that HFD had no significant effect on the liver level of TCH in model group as compared with control. No statistical difference of TCH and FFAs was observed between model and AEE groups.

**Table 1:** Effects of AEE on lipid profile in hepatic tissue from hyperlipidemic rat

Variables	Control	Model	AEE
TG	3.54 ± 1.30^**^	8.02 ± 1.26	5.91 ± 0.98^**##^
TCH	6.69 ± 3.57	8.32 ± 4.25	9.12 ± 1.95
LDL	4.44 ± 1.19^**^	7.61 ± 1.30	6.02 ± 1.41^*#^
FFAs	11.02 ± 2.27^**^	18.63 ± 1.90	17.21 ± 2.67^##^

Data were expressed as mean ± SD. FFAs: free fatty acids; ^*^ *P* < 0.05, ^**^ *P* < 0.01 compared with the model group; ^#^ *P* < 0.05, ^##^ *P* < 0.01 compared with the control group. The units of TG, TCH, LDL and FFAs were mmol/g liver

### Histopathology observation

Pathological results of liver, stomach and duodenum were shown in Additional file 4. Liver histological examinations of control showed normal cell architecture, while significant changes were observed in model group. Serious fatty degenerations of liver cells in model group were found and pointed with black arrow (Arrow 1). In comparison with the model group, the fatty degenerations in AEE treatment group were significantly decreased and the representative results were shown (Arrow 2). No histopathological changes in stomach and duodenum were observed from the rats in control and model groups. However, in AEE group, a few evidences of hyperemia and edema of lamina propria of stomach and duodenum and the ecclasis of gastric mucosa could be found in rats (Arrow 3–5).

**Fig. 2:**
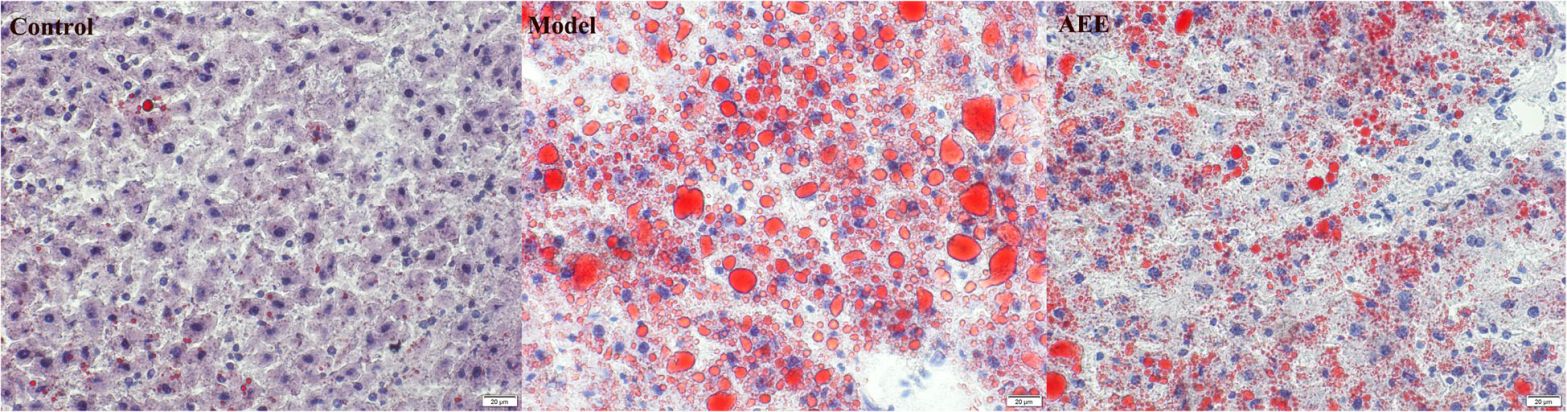
Representative images of rat hepatic tissue stained with oil red O (× 400)

Oil red O staining from frozen section was applied to observe the lipid accumulation in the rat liver (Fig. 2). The results indicated that few lipid droplets were observed in the normal rats, while lots of lipid droplets were presented in the model group suggesting the massive lipid accumulation in the liver. In AEE group, HFD-induced lipid accumulation was remarkably improved by AEE treatment. The liver results of HE and oil red O staining were in agreement with each other indicating the ameliorative effects of AEE on lipid accumulation in liver.

### Effect of AEE on liver tissue metabolomics

**Fig. 3:**
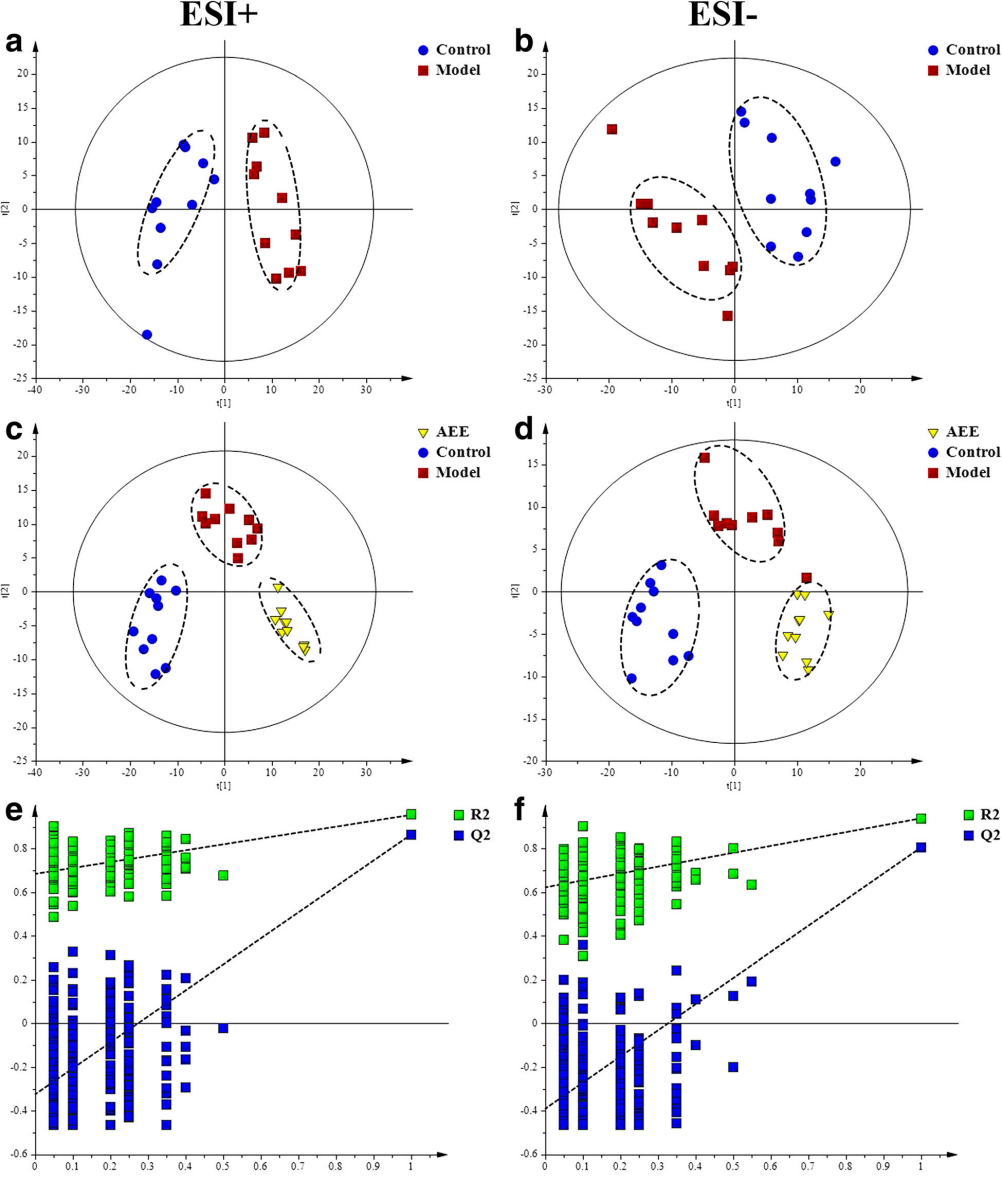
Score plots and permutation test derived from the liver tissue of rats in different group. **a** and **b**, PCA score plots between control and model groups; **c** and **d**, PLS-DA score plots among control, model and AEE groups; **e** and **f**, permutation test from PLS-DA models

Representative total ion chromatograms (TICs) of the liver tissue samples analyzed by UPLC-Q-TOF/MS were shown in Additional file 5. In ESI+ and ESI-, the TICs showed good separations and strong sensitivity of the established method. PCA was employed to find out the metabolic distinction between control and model groups. As shown by the score plots in Fig. 3a and b, the metabolic profiles of liver tissue in ESI+ and ESI- from the control and model groups were clearly separated, in which the samples in model group exhibited a tendency to be away from those in control. These results indicated that there was significant difference in liver metabolite profiles from the two groups. Model parameter R^2^X represents the explanative ability of the model. The R^2^X of PCA models in ESI+ and ESI- were 0.612 and 0.654, indicating the data can be highly elucidated by the two models.

PLS-DA, a supervised multivariable statistical method, was conducted to further assess the influence of AEE on metabolic pattern. Score plots of the PLS-DA model were shown in Fig. 3c and d (ESI+: R^2^X = 0.529, R^2^Y = 0.966, Q^2^ = 0.879; ESI-: R^2^X = 0.589, R^2^Y = 0.955, Q^2^ = 0.844). All samples fell inside the 95% confidence interval, which was represented by an ellipse in Fig. 3c and d. In both of the ESI+ and ESI-, the control, model and AEE groups were classified clearly. The metabolic profile of rats in AEE treated group fairly differed from the model group and closed to the control, indicating the deviations induced by HFD were significantly improved after AEE treatment. These results were in accordance with the results of blood lipids analysis and observation of pathological changes. Validation with 200 random permutation tests generated intercepts of R^2^ = 0.69 and Q^2^ = −0.29 from positive model data (Fig. 3e) and R^2^ = 0.64 and Q^2^ = −0.36 from negative model data (Fig. 3f). These results indicated that the PLS-DA models had good predictive ability and were robust without overfitting.

**Fig. 4:**
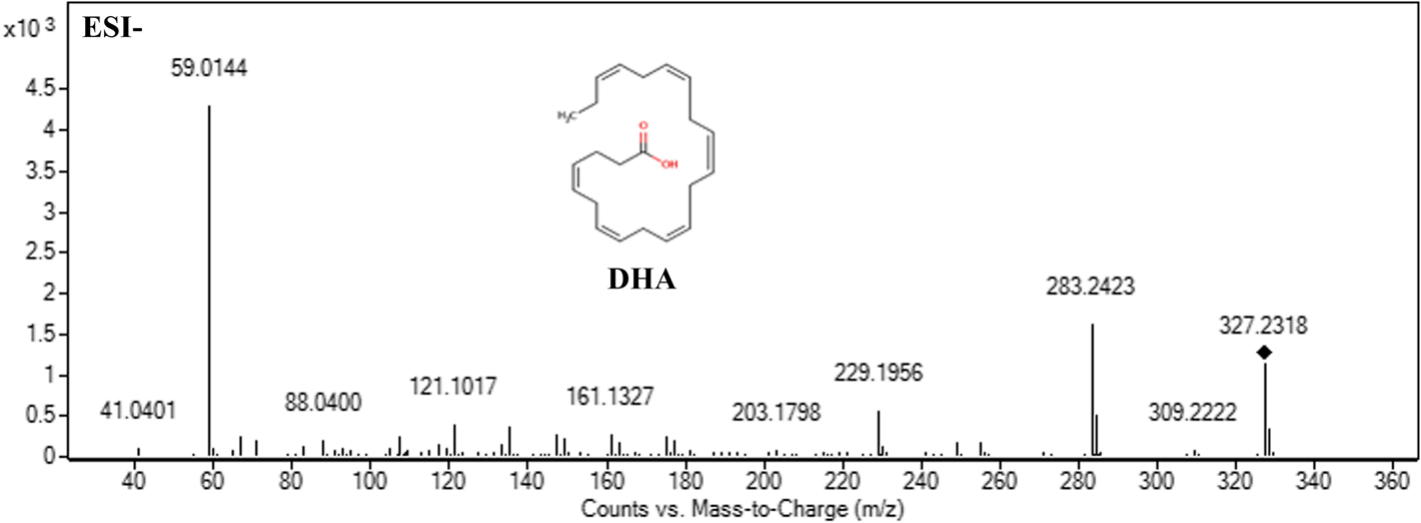
Mass spectra of biomarker at m/z 327.2352 in negative ion mode (Collision energy: 20 eV)

Among the ions, with VIP values above 1 and *P*-values below 0.05, 29 metabolites were selected and identified as potential biomarkers (Table 2). Here, a potential biomarker with m/z 327.2352 in negative mode is taken as an example to illustrate the identification process. First, we search the HMDB or METLIN database by using accurate mass. The ion of m/z 327.2352 was searched by comparing the extract mass with those enrolled in the HMDB and METLIN database. The result showed that some possible candidates matching with the accurate mass (mass difference lower than 10 ppm). Then, the fragments of these candidates in negative ion mode were investigated. Some major fragment ions including 59.0143, 121.1017, 161.1327, 229.1956 and 283.2423 (shown in Fig. 4) were matched with the fragmentation pattern of docosahexaenoic acid (DHA). Finally, the ion of m/z 327.2352 was tentatively identified as DHA. Fragments of the potential biomarkers matched in HMDB or METLIN databases in metabolites identification were shown in Additional file 6.

**Table 2:** Potential biomarkers in liver associated with AEE treatment based on UPLC-Q-TOF/MS analysis in hyperlipidemic rat

NO.	RT	VIP	m/z	Formula	Metabolite	Trend	
						M/C	AEE/M
1	1.11	2.33	118.0866	C_5_H_11_NO_2_	Valine	↑^**^	↑
2	1.73	2.46	137.0459	C_5_H_4_N_4_O	Hypoxanthine	↑^**^	↓^*^
3	2.85	3.38	166.0857	C_9_H_11_NO_2_	Phenylalanine	↓	↓^*^
4	9.31	1.89	466.3179	C_26_H_43_NO_6_	Glycocholic acid	↑	↑^**^
5	12.12	2.76	524.3730	C_26_H_54_NO_7_P	LysoPC (18:0)	↓	↓^**^
6	13.63	2.92	568.3413	C_30_H_50_NO_7_P	LysoPC (22:6)	↓^**^	↓
7	13.81	6.30	544.3411	C_28_H_50_NO_7_P	LysoPC (20:4)	↓^**^	↓ ^*^
8	14.83	2.087	400.3437	C_23_H_45_NO_4_	Palmitoylcarnitine	↑^**^	↓
9	16.91	2.15	428.3752	C_25_H_49_NO_4_	Stearoylcarnitine	↑^**^	↑
10	1.74	1.85	132.1017	C_6_H_13_NO_2_	Isoleucine	↓^*^	↑
11	3.41	1.44	220.1178	C_9_H_17_NO_5_	Pantothenic acid	↓^**^	↑
12	3.93	1.52	205.0971	C_11_H_12_N_2_O_2_	Tryptophan	↓	↓^**^
13	10.32	1.82	318.301	C_18_H_39_NO_3_	Phytosphingosine	↓^**^	↑^**^
14	11.08	1.81	450.3228	C_26_H_43_NO_5_	GUDCA	↑	↑^**^
15	13.35	1.36	494.3256	C_24_H_48_NO_7_P	LysoPC (16:1)	↑^*^	↑^**^
16	14.04	1.41	424.3438	C_25_H_45_NO_4_	Linoleyl carnitine	↑^**^	↓
17	1.22	1.08	147.0767	C_5_H_10_N_2_O_3_	Glutamine	↑^**^	↓
18	1.66	1.16	613.1611	C_20_H_32_N_6_O_12_S_2_	Oxidized glutathione	↓	↑^**^
19	1.67	1.13	123.0554	C_6_H_6_N_2_O	Niacinamide	↑	↑^**^
20	9.90	1.10	500.3053	C_26_H_45_NO_6_S	TCDCA	↓^**^	↑
21	14.14	1.26	542.3231	C_28_H_48_NO_7_P	LysoPC (20:5)	↑	↓
22	15.16	3.23	546.3561	C_28_H_52_NO_7_P	LysoPC (20:3)	↑^**^	↓
23	1.75	2.10	267.0754	C_10_H_12_N_4_O_5_	Inosine	↑^**^	↓^**^
24	4.68	2.20	784.1549	C_27_H_33_N_9_O_15_P_2_	FAD	↑^**^	↓
25	16.66	2.42	327.2352	C_22_H_32_O_2_	DHA	↓^*^	↓
26	16.91	3.04	303.2348	C_20_H_32_O_2_	Arachidonic acid	↓^*^	↑
27	1.10	1.00	195.0525	C_6_H_12_O_7_	Gluconic acid	↑^**^	↓
28	4.59	1.01	686.1462	C_21_H_35_N_7_O_13_P_2_S	Dephospho-CoA	↑	↓^**^

M/C: model versus control; AEE/M: AEE versus model; ^*^ *P* < 0.05, ^**^ *P* < 0.01. GUDCA: Glycoursodeoxycholic acid; TCDCA: Taurochenodesoxycholic acid; DHA: Docosahexaenoic acid; FAD: flavine-adenine dinucleotide

**Fig. 5:**
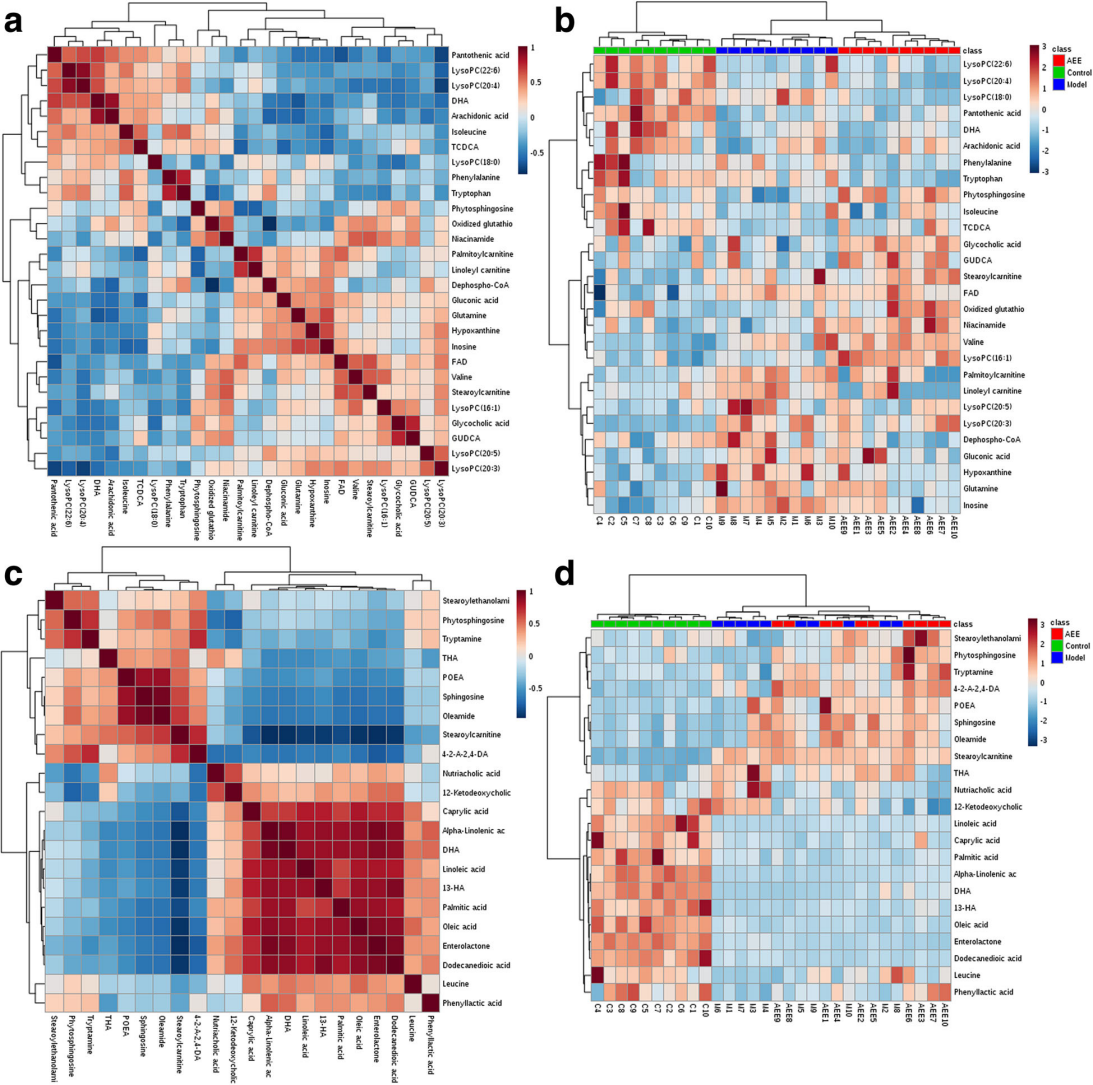
Correlation analysis and clustering heatmap of potential biomarkers in feces and liver. Increasing expression values are coded with green to red colors. Rows indicate potential biomarkers; columns indicate samples in different groups. **a** and **b**: Pearson’s correlations and clustering heatmap of the metabolites determined from liver samples; **c** and **d**: Pearson’s correlations and clustering heatmap of the metabolites determined from fecal samples

To further understand the metabolic differences, the identified biomarker data were analyzed using correlation analysis and clustering heatmap. Identified biomarkers were systematically searched for Pearson’s correlations, and the correlations were indicated in different colors (Fig. 5a). Identified metabolites were visualized in a clustering heatmap which illustrated the relative increase (red) or decrease (green) values (Fig. 5b). Compared with the metabolic profiles of HFD-fed rats, 12 metabolites were increased and 16 decreased in AEE treated rats. For instance, valine, isoleucine, glycocholic acid, LysoPC (16:1) and arachidonic acid were showed an increasing trend in AEE group; In contrast, hypoxanthine, phenylalanine, LysoPC (18:0), LysoPC (20:4), tryptophan, inosine and dephospho-CoA were decreased. These metabolites were mainly involved in amino acid metabolism, bile acid metabolism, fatty acid metabolism and glycerophospholipid metabolism.

### Effect of AEE on fecal metabolomics

**Fig. 6:**
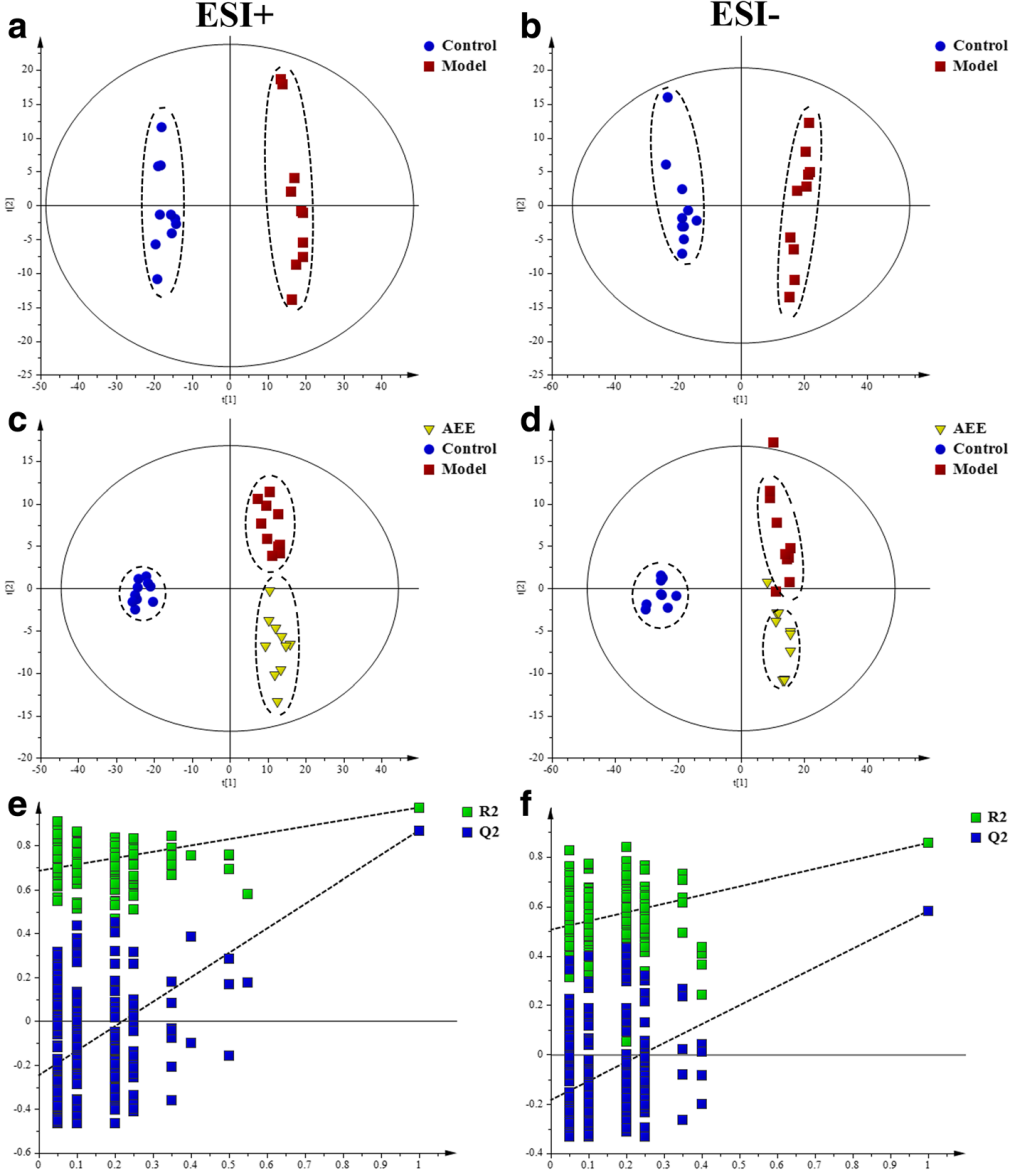
Score plots and permutation test derived from the feces of rats in different group. **a** and **b**, PCA score plots between control and model groups; **c** and **d**, PLS-DA score plots among control, model and AEE groups; **e** and **f**, permutation test from PLS-DA models

Fecal metabolomics presented similar results with that of liver tissue, suggesting the ameliorative effects of AEE in hyperlipidemic rats. Additional file 7 showed typical TICs of fecal samples in positive and negative modes. Detail metabolomics differences between control and model groups were revealed by PCA models in Fig. 6a and b (ESI+: R^2^X = 0.601; ESI-: R^2^X = 0.643). According to the score plots, samples of model group were clustered away from those of control group, indicating that hyperlipidemia was successfully induced.

In Fig. 6c and d, clear separations were observed among control, model and AEE groups in both of positive and negative modes. The score plots of PLS-DA showed that the AEE treated group clustered and deviated from model group, which suggested that AEE treatment partially recovered the hyperlipidemia state. The permutation test was also carried out to test the over-fitting of PLS-DA after modeling the data, and the results (intercepts of ESI+: R^2^ = 0.68, Q^2^ = −0.26; intercepts of ESI-: R^2^ = 0.50, Q^2^ = −0.19) demonstrated that the original PLS-DA models were robust without overfitting (Fig. 6e and f).

The candidate metabolites with VIP > 1 and *P* < 0.05 were selected as potential biomarkers. Following the criteria above, a total of 22 endogenous metabolites in feces were selected. The fragments of selected metabolites in MS/MS analysis matched in HMDB or Metlin databases were listed in Additional file 8. The detail alteration trends of biomarkers were shown in Table 3. The set of identified metabolites was searched through for Pearson’s correlations, and each metabolite was used to construct a heat map. The results of correlation analysis and clustering heatmap were summarized in Fig. 5c and d. In AEE group, the metabolites such as phytosphingosine, sphingosine, linoleic acid, tryptamine, oleamide and caprylic acid were increased in comparison with the model group. However, some metabolites including nutriacholic acid, leucine, THA and oleic acid were decreased.

**Table 3:** Potential biomarkers in feces associated with AEE treatment based on UPLC-Q-TOF/MS analysis in hyperlipidemic rat

NO.	RT	VIP	m/z	Formula	Metabolite	Trend	
						M/C	AEE/M
1	6.8	6.04	391.2835	C_24_H_38_O_4_	Nutriacholic acid	↑	↓
2	6.92	2.82	318.2996	C_18_H_39_NO_3_	Phytosphingosine	↑	↑^*^
3	7.53	4.67	300.2892	C_18_H_37_NO_2_	Sphingosine	↑^*^	↑
4	10.19	4.80	281.2468	C_18_H_32_O_2_	Linoleic acid	↓^**^	↑
5	13.25	3.27	428.3733	C_25_H_49_NO_4_	Stearoylcarnitine	↑^**^	↑
6	1.74	1.71	132.1011	C_6_H_13_NO_2_	Leucine	↓^*^	↓
7	3.55	1.77	161.1061	C_10_H_12_N_2_	Tryptamine	↑	↑
8	7.54	2.20	282.2788	C_18_H_35_NO	Oleamide	↑^*^	↑
9	0.67	1.51	145.1211	C_8_H_16_O_2_	Caprylic acid	↓^**^	↑
10	6.76	1.43	298.2731	C_18_H_35_NO_2_	POEA	↑	↑
11	11.15	1.63	279.2312	C_16_H_32_O_2_	Palmitic acid	↓^**^	↑
12	16.55	1.52	328.3206	C_20_H_41_NO_2_	Stearoylethanolamide	↑	↑
13	12.03	1.17	357.2785	C_24_H_36_O_2_	THA	↑^**^	↓^*^
14	3.73	1.45	206.0449	C_10_H_9_NO_4_	4-(2-A)-2,4-DA	↑^*^	↑
15	4.36	1.74	165.0550	C_9_H_10_O_3_	Phenyllactic acid	↓^**^	↑^*^
16	5.41	2.25	297.1122	C_18_H_18_O_4_	Enterolactone	↓^**^	↓
17	11.5	4.69	299.2580	C_18_H_36_O_3_	13-HA	↓^**^	↑
18	13.23	1.51	277.2163	C_18_H_30_O_2_	Alpha-Linolenic acid	↓^**^	↑
19	16.43	2.75	281.2475	C_18_H_34_O_2_	Oleic acid	↓^**^	↓
20	4.04	1.23	229.1435	C_12_H_22_O_4_	Dodecanedioic acid	↓^**^	↓
21	7.68	1.05	389.2685	C_24_H_38_O_4_	12-Ketodeoxycholic acid	↓	↓
22	13.87	1.24	327.232	C_22_H_32_O_2_	DHA	↓^**^	↑

M/C: model versus control; AEE/M: AEE versus model; ^*^ *P* < 0.05, ^**^ *P* < 0.01. POEA: Palmitoleoyl ethanolamide; THA: Tetracosahexaenoic acid; 4-(2-A)-2,4-DA: 4-(2-Aminophenyl)-2,4-dioxobutanoic acid; DHA: Docosahexaenoic acid; 13-HA: 13-hydroxyoctadecanoic acid

### Effect of AEE on metabolic pathway analysis

**Fig. 7:**
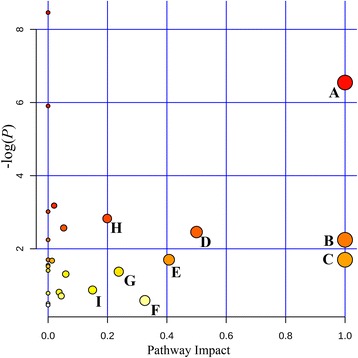
Disturbed pathways in response to hyperlipidemia and AEE treatment. **a**, Valine, leucine and isoleucine biosynthesis; **b**, Linoleic acid metabolism; **c**, alpha-Linolenic acid metabolism; **d**, Phenylalanine, tyrosine and tryptophan biosynthesis; **e**, Phenylalanine metabolism; **f**, Arachidonic acid metabolism; **g**, Nicotinate and nicotinamide metabolism; **h**, Tryptophan metabolism; I, Alanine, aspartate and glutamate metabolism

**Fig. 8:**
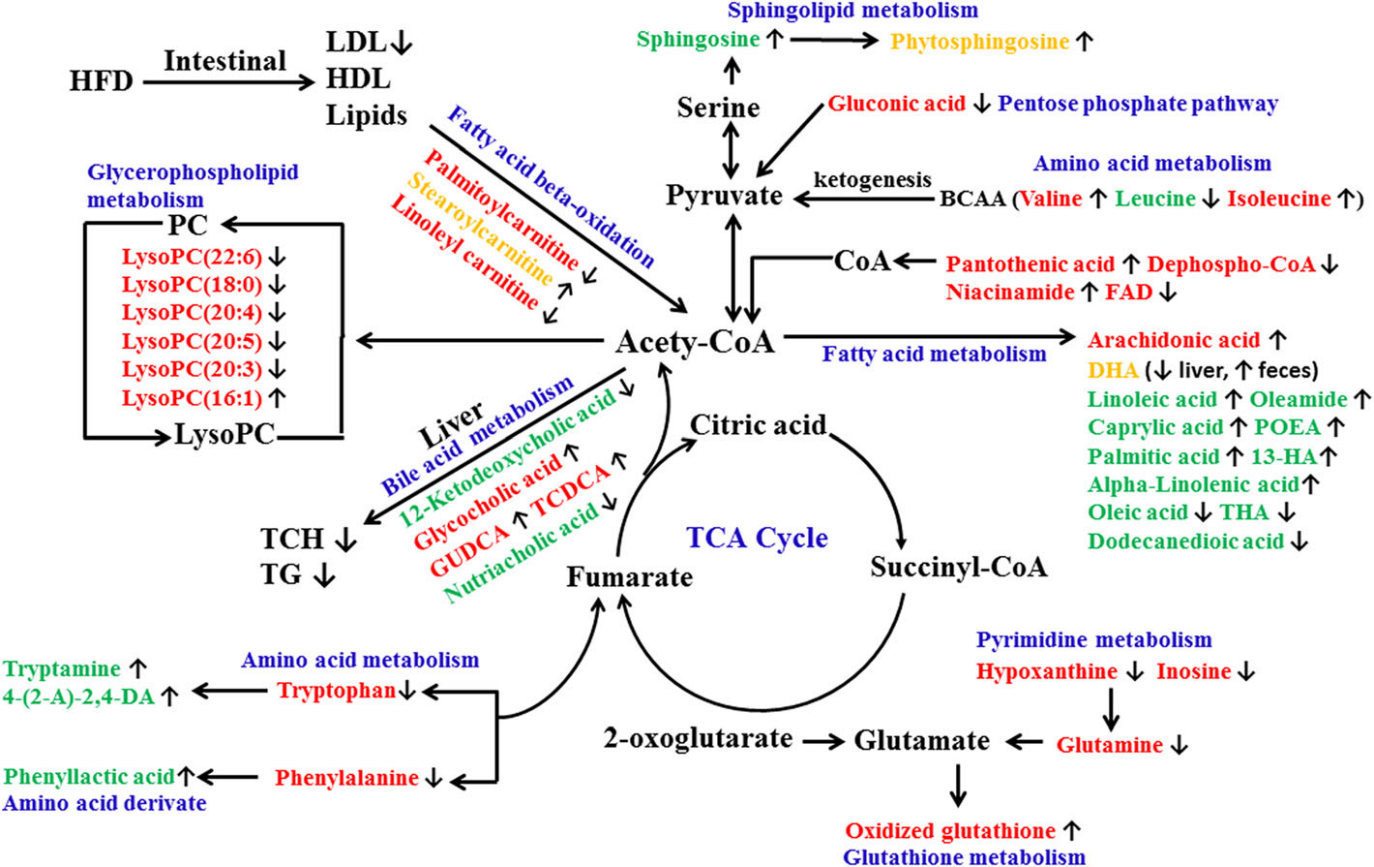
Potential metabolic pathways disturbed in hyperglycemic rat induced by HFD and alterations by AEE treatment. Metabolite names in green or red indicated they were found in feces or liver, respectively, and the metabolites in orange were found in both. The names of the possible metabolic pathways were denoted by words in blue. After AEE treatment, increased trend was indicated by↑and decreased trend was indicated by↓

To identify and visualize the affected metabolic pathways in response to AEE treatment in hyperlipidemic rats, the detailed pathways analysis was performed by MetaboAnalyst 3.0 which was a free, web-based tool that could help researchers to identify the most relevant pathways involved in the conditions under study. In present study, the HMDB IDs of metabolites found in feces and liver and *Rattus norvegicus* (rat, 81 pathways) pathway library were selected for pathway analysis. The impact-value threshold was set to 0.10 and the pathway with impact-value above this threshold was filtered out. The results revealed that the primary disturbed pathways were valine, leucine and isoleucine biosynthesis, tryptophan metabolism, phenylalanine, tyrosine and tryptophan biosynthesis, linoleic acid metabolism, phenylalanine metabolism, alpha-linolenic acid metabolism, nicotinate and nicotinamide metabolism, alanine, aspartate and glutamate metabolism and arachidonic acid metabolism (Additional file 9). Figure 7 showed the summary of the pathway analysis, and an integrative view plot of metabolites was shown in Fig. 8.

## Discussion

As a promising drug candidate, AEE reduce the side effects of aspirin and eugenol, and can generate synergistic effects to improve therapeutic effects. Results of pharmacodynamics experiments revealed that AEE carried many biological activities, including anti-inflammation, anti-thrombosis, anti-oxidation, analgesia and antipyresis [5, 15, 16]. In our previous study, AEE treatment could regulate the disorders of serum lipid in hyperlipidemic rat model fed with HFD [10, 17]. In this study, the lipid profile in liver was investigated by oil red O staining and lipid analysis. The results showed that the hyperlipidemia model was successfully established in rat after a continuous administration of HFD for 8 weeks. Meanwhile, these results also indicated the HFD-fed rats had developed a metabolic disorder of lipid in liver. AEE treatment had a significant effect on lipid metabolism disorders such as the reducing of lipid droplets, LDL and TG, which could be also verified by the metabolomics analysis of liver and feces in score plots.

The present study demonstrated that 28 identified potential biomarkers in liver and 22 in feces were relevant with the disturbance of the metabolism in hyperlipidemic rats, which were mainly involved in glycerophospholipid metabolism, amino acid metabolism, fatty acid metabolism, sphingolipid metabolism, purine metabolism, bile acid metabolism and glutathione metabolism. Furthermore, it was confirmed that AEE treatment could show favorable inhibitation on hyperlipidemia, which might be associated with the efficacy of AEE by restoring alterations of some biomarkers found in this research.

Lysophosphatidylcholines (LysoPCs) are regularly produced from the hydrolysis of oxidized phosphatidylcholine in LDL by phospholipase A2, and play various roles in many important biological processes. As immediate-response molecules, LysoPCs can trigger inflammation, cyclooxygenase expression and the autoimmune response independently or through activation of specific G-protein-coupled receptors [18]. It has been suggested that LysoPCs are associated with many diseases, including atherosclerosis, sepsis, diabetes and cancer [19]. Accumulating evidences suggest that hyperlipidemia refers to a condition where there is an imbalance of LysoPCs profiling. For example, it has been reported that LysoPC (22:6) and LysoPC (20:4) showed a significant decrease in mice fed with HFD for 4 weeks, and the levels of LysoPC (16:1) and LysoPC (20:5) were increased in atherosclerosis rabbit [20–22]. In present study, three LysoPCs in rat fed with HFD were down-regulated and three LysoPCs were up-regulated. The changing trends of these LysoPCs were matched with above reports. In hyperlipidemic rat, these LysoPCs appeared different from that in control rat, which indicated that the disorder of glycerophospholipid metabolism might be relevant to the pathogenesis of hyperlipidemia. AEE treatment showed favorable regulation on the variation of these LysoPCs such as the decrease of LysoPC (20:5) and LysoPC (20:3), indicating that the effects of AEE on glycerophospholipid metabolism might contribute to its anti-hyperlipidemia efficacy. These metabolism changes of LysoPCs are helpful to improve our understanding of the potential mechanism of AEE.

Carnitine, a vitamin-like compound synthesized in the liver, kidneys and brain, is an essential factor in fatty acid metabolism in mammals. The most important known metabolic function of carnitine is to transport fat into the mitochondria of cells for fatty acid oxidation. Palmitoylcarnitine, stearoylcarnitine and linoleyl carnitine are long-chain acyl fatty acid derivative ester of carnitine. In this study, palmitoylcarnitine, stearoylcarnitine and linoleyl carnitine were markedly increased in model group than those in the control. Song et al. reported that energy metabolism impairment was a character of hyperlipidemia that glycolysis and glucose aerobic oxidation were inhibited and the fatty acid oxidation was promoted in hyperlipidemic rat [23]. The energy metabolism impairment was also partly proved by the accumulation of gluconic acid, which might be resulted from the inhibitation of glucose metabolism. Meanwhile, it was also reported that palmitoylcarnitine and linoleyl carnitine were significantly increased in patients with liver disease such as nonalcoholic fatty liver disease (NAFLD) and chronic liver failure [24, 25]. HFD could destroy the equilibrium between the formation and degradation of lipid, and lead to excessive lipid deposition in hepatocytes, resulting disorder of liver function. It is presumed that the disturbed energy metabolism and the long time impairment of liver function may result in the increase of palmitoylcarnitine, stearoylcarnitine and linoleyl carnitine. The increase of palmitoylcarnitine, linoleyl carnitine and gluconic acid could be inhibited by AEE treatment, suggesting the efficacy of AEE might ascribe to the regulation of fatty acid metabolism and liver function.

Docosahexaenoic acid (DHA) is a long-chain polyunsaturated fatty acid. In the present study, levels of DHA in liver and feces were decreased compared with the control rat, which agreed with the previous reports that DHA was decreased in patients with liver cirrhosis [26]. It was speculated that pathological changes of fatty liver was the reason for the decreased DHA. Many studies have demonstrated the positive effects of dietary DHA on cardiovascular health, which DHA could reduce inflammation and total body fat and attenuate dyslipidemia [27]. The level of DHA in feces of AEE group was higher than that in the model group, which might be beneficial for the improvement of blood lipids. Arachidonic acid (AA) is a biological active n-6 polyunsaturated fatty acid, which could be used to produce leukotrienes and prostaglandins. Related evidence has confirmed that patients with cardiovascular disease have lower AA concentration than normal population, and the reduction of AA level may contribute to cardiovascular risks [28]. In present work, the levels of AA were decreased in model group compared with the normal control group, which was consistent with the findings by Gu et al. [29]. One possible explanation might be that AA was consumed to enhance cardiovascular protection in hyperlipidemic rat. AEE treatment could successfully reverse the reduction of AA, suggesting that AA might be one potential biomarker of the lipid regulation by AEE.

In this work, the levels of linoleic acid, caprylic acid, palmitic acid and alpha-linolenic acid were significantly lowered by HFD in feces. The findings of the decreased levels of these potential biomarkers in feces of hyperlipidemic rat were partly consistent with the observation by Sham et al. [30]. Moreover, some lectures have reported that caprylic acid, linoleic acid and alpha-linolenic acid can reduce the hepatic and plasma lipid levels in HFD-fed animals [31–33]. Interestingly, the reduction of linoleic acid, caprylic acid, palmitic acid and alpha-linolenic acid could be up-regulated by the treatment of AEE, indicating AEE could ameliorate the disturbed fatty acid metabolism. The levels of oleamide, oleic acid and dodecanedioic acid were also affected by HFD, and AEE treatment had regulating effect on these metabolites involved in fatty acid metabolism. Few previous reports on MS-based fecal metabonomic studies have been performed to reveal the complex interactions between fatty acid metabolism and hyperlipidemia. Underlying causes of the alteration of the metabolites remain unclear and may be of considerable interest in future studies.

Amino acid metabolism is affected by many factors such as energy metabolism and liver function. Valine, phenylalanine, isoleucine, tryptophan and glutamine and leucine involved amino acid metabolism were identified and selected as biomarkers associated with hyperlipidemia. Branched-chain amino acids (BCAAs) include leucine, isoleucine, and valine, which are three of the nine essential amino acids. BCAAs have been reported to attenuate HFD-induced weight gain, decrease fat mass, inhibit hepatic lipogenic enzymes and reduce hepatic triglyceride content [34]. In AEE group, increased levels of valine and isoleucine and decreased leucine were observed in contrast to model group, which were partly consistent with previous report associated with anti-hyperlipidemia effect [35]. It might be inferred that AEE treatment suppressed the ketogenesis to lead the elevated levels of valine and isoleucine. Compared with the control group, the levels of glutamine was higher in liver of model group. According to the previous reports, HFD could lead the increase of glutamine in hyperlipidemic rat, suggesting that glutamine might be an important amino acid during the progression of hyperlipidemia [36]. Elevated glutamine in the liver indicates the increase of energy storage, which may be the results from HFD consumption [37]. Glutamine is also the precursors of the major natural antioxidant glutathione, thus, variation of glutamine is supposed to exert influence on glutathione metabolism, which consequently leads to oxidative injures. It is noteworthy that the model group showed the decreased level of oxidized glutathione, which might indicate the disruption of the antioxidative system. The down-regulation of glutamine and up-regulation of oxidized glutathione after AEE treatment may suggest the recovery of energy metabolism and the improvement of oxidative stress. The decrease in phenylalanine and tryptophan levels in liver of HFD-fed rats might be the manifestation of abnormal liver function. Pathological study confirmed that liver injuries were alleviated by AEE treatment, which might contribute to restoring liver function. However, no significant changes were observed in the levels of phenylalanine and tryptophan between model and AEE groups. Further studies are needed to explore the reducing effect of AEE on phenylalanine and tryptophan in hyperlipidemic rat.

Bile acids are physiological detergents produced in liver and play an important role in cholesterol metabolism, which can facilitate the excretion, absorption, and transport of fats and sterols in intestine and liver. It was found that increased total bile acid level is responsible for the anti-hyperlipidemia effect [38]. As a secondary bile acid,glycocholic acid and glycoursodeoxycholic acid (GUDCA) were produced by the action of enzymes existing in the microbial flora of the colonic environment. Rats in the model group with longtime of HFD showed elevated levels of glycocholic acid and GUDCA, which might prove the promotion the bile acid biosynthesis. With the application of LC-MS/MS method, Liu et al. reported that increased concentration of glycocholic acid in plasma and liver is contributed to cholesterol eliminating [39]. It was also reported that a significant decrease in the molar percentages of GUDCA was observed in the patients with heterozygous familial hypercholesterolemia compared to the corresponding values in the controls [40]. In present study, sharp increased level of glycocholic acid and GUDCA in liver was observed after AEE treatment compared with the model group, which could promote the transformation of cholesterol in liver and further enhance the lipid level decrease. Taurochenodesoxycholic acid (TCDCA) is formed by conjugation of chenodeoxycholate with taurine in the liver. In hyperlipidemia rat, large amount of cholesterol is accumulated in liver, and then cause damage of liver function. The decreased concentration of TCDCA in model group might be associated with the disorder of liver function. AEE treatment reduced lipid accumulation and restored liver function, which may be the reasons for the increased TCDCA in AEE group. The results of glycocholic acid, GUDCA and TCDCA suggested that AEE could ameliorate the disturbed bile acid metabolism in hyperlipidemia rat.

Inosine is a purine nucleoside, which is an intermediate in the degradation of purines and in pathways of purine salvage. Hypoxanthine is a naturally occurring purine derivative and a reaction intermediate in the metabolism of adenosine. The increased levels of inosine and hypoxanthine in the liver of the model group were found in this work, which implied the enhancement of purine metabolism. This is in good agreement with the previous observation that HFD could cause concurrent elevations of inosine and hypoxanthine in rat myocardium and testicles in the preobesity state with the application of NMR-based metabonomic approach [41]. After AEE treatment, the increased levels of inosine and hypoxanthine were down-regulated, indicating AEE had regulation effect on purine metabolism.

Sphingosine and phytosphingosine are the major bases of the sphingolipids in mammals, which are involved in many cell processes including cell-cell interaction, cell proliferation, cell growth and apoptosis. It has been reported that phytosphingosine levels in plasma samples from hyperlipidemic rat are higher than that in the control rat [42]. Significant increase in sphingolipids may reduce reverse cholesterol transport pathway, which may be one of the reasons for the lipid disorder in hyperlipidemia-related diseases [43, 44]. In this study, increased levels of sphingosine and phytosphingosine in feces in HFD-induced hyperlipidemic rat were observed, suggesting the promotion of sphingolipid metabolism under hyperlipidemia condition. However, the increased levels of sphingosine and phytosphingosine were not down-regulated in feces after AEE treatment. It is noted that the level of phytosphingosine was significantly decrease in liver samples, whereas AEE treatment reversed this trend. There is increasing evidence that obvious decrease of phytosphingosine is observed in patients with diabetic nephropathy and type 2 diabetes mellitus, which may be influenced by glucosylceramide and glucose metabolism [45, 46]. Different trends of phytosphingosine were found in feces and liver in this study, suggesting the disturbance of sphingolipid metabolism. Further researches are required to reveal the physiological roles of phytosphingosine and sphingosine in hyperlipidemia.

Pantothenic acid is needed to form coenzyme-A (CoA), which is critical for the lipid metabolism. The concentration of pantothenic acid in the liver was significantly lower in the model group than the control group. Kei et al. reported that HFD could increase the utilization of pantothenic acid through the increase preservation of fatty acid for the synthesis of the body weight [47]. In contrast, the level of pantothenic acid was noticed to be increased in AEE group than the model, which might be one of the reasons for the reduced body weight in hyperlipidemic rat reported in our previous study [10].

It is well known that that hyperlipidemia is a multi-factorial disease. Many etiological factors including inflammation, oxidative stress, energy metabolism imbalance and endothelial dysfunction could lead the development of hyperlipidemia. As a new chemical drug for hyperlipidemia treatment, more studies are needed to investigate the underlying action mechanism of AEE in future studies.

## Conclusion

In this study, anti-hyperlipidemia effect of AEE was confirmed by lipid analysis and metabonomic approach. UPLC-Q-TOF/MS-based liver and feces metabonomic studies had revealed the regulation effects of AEE on hyperlipidemic rat. According to the identified metabolites, it was found that AEE could partially recover the metabolic alternations induced by hyperlipidemia via the possible metabolic pathways: glycerophospholipid metabolism, amino acid metabolism, fatty acid metabolism, sphingolipid metabolism, purine metabolism, bile acid metabolism and glutathione metabolism. Our findings might provide pharmacological basis of AEE against hyperlipidemia, and indicated that AEE could be developed into an agent for hyperlipidemia treatment.

## Additional files

Additional file 1: Results of blood lipids after high fat diet administrated for 8 weeks. (PDF 60 kb)

Additional file 2: Optimized gradient elution program of UPLC-Q-TOF/MS in fecal and liver tissue metabonomic studies. (PDF 44 kb)

Additional file 3: Effects of AEE on blood lipid levels in hyperlipidemic rats (*n* = 10). (PDF 44 kb)

Additional file 4: Histopathological results of liver, stomach and duodenum after a five-week AEE treatment (HE × 100). (PDF 420 kb)

Additional file 5: Typical UPLC-Q-TOF/MS total ion chromatograms (TICs) of liver tissue in positive and negative ion modes. (PDF 260 kb)

Additional file 6: Fragments matched in HMDB or Metlin databases in metabolites identification in liver tissue. (PDF 49 kb)

Additional file 7: Typical UPLC-Q-TOF/MS total ion chromatograms (TICs) of feces in positive and negative ion modes. (PDF 242 kb)

Additional file 8: Fragments matched in HMDB or Metlin databases in metabolites identification in feces. (PDF 52 kb)

Additional file 9: Pathway analysis result with MetaboAnalyst 3.0. (PDF 43 kb)

## Abbreviations

13-HA: 13-hydroxyoctadecanoic acid; FFAs: free fatty acids
4-(2-A)-2,4-DA: 4-(2-Aminophenyl)-2,4-dioxobutanoic acid
AEE: aspirin eugenol ester
CMC-Na: Carboxymethylcellulose sodium
CVD: Cardiovascular disease
DHA: Docosahexaenoic acid
ESI: electrospray ionization
FAD: flavine-adenine dinucleotide
GUDCA: Glycoursodeoxycholic acid
HDL: high-density lipoprotein
HE: hematoxylin and eosin
HFD: high fat diet
LDL: low-density lipoprotein
PCA: principal component analysis
PLS-DA: partial least squares discriminant analysis
POEA: Palmitoleoyl ethanolamide
TCDCA: Taurochenodesoxycholic acid
TCH: total cholesterol
TG: triglycerides
THA: Tetracosahexaenoic acid
TIC: total ion chromatograms
VIP: variance importance for projection
