# Supplementary material for: Feces and liver tissue metabonomics studies on the regulatory effect of aspirin eugenol eater in hyperlipidemic rats
Source: Lipids Health Dis. 2017 Dec 11;16:240. doi: 10.1186/s12944-017-0633-0 (PMC5725792; doi:10.1186/s12944-017-0633-0)
Supplement: Supplementary file 1 — Results of blood lipids after high fat diet administrated for 8 weeks. (PDF 60 kb) [file 12944_2017_633_MOESM1_ESM.pdf]

Additional file 1: Results of blood lipids after high fat diet administrated for 8 weeks.

Variables	Feed	TG	TCH	HDL	LDL
Group I	Standard feed	0.41 ± 0.07	1.15 ± 0.11	0.55 ± 0.07	0.19 ± 0.06
Group II	HFD	0.54 ± 0.11**	1.48 ± 0.08**	0.40 ± 0.06**	0.48 ± 0.08**

HFD: high fat diet; TG: triglyceride; HDL: high density lipoprotein; LDL: low density lipoprotein; TCH: total cholesterol. The units of TG, TCH, HDL and LDL were mmol/L; $^{**}p < 0.01$ significant difference from Group I. All data were shown as mean ± SD.
