# Supplementary material for: Feces and liver tissue metabonomics studies on the regulatory effect of aspirin eugenol eater in hyperlipidemic rats
Source: Lipids Health Dis. 2017 Dec 11;16:240. doi: 10.1186/s12944-017-0633-0 (PMC5725792; doi:10.1186/s12944-017-0633-0)
Supplement: Supplementary file 2 — Optimized gradient elution program of UPLC-Q-TOF/MS in fecal and liver tissue metabonomic studies. (PDF 44 kb) [file 12944_2017_633_MOESM2_ESM.pdf]

Additional file 2: Optimized gradient elution program of UPLC-Q-TOF/MS in fecal and liver tissue metabonomic studies.

Time	Feces		Time	Liver tissue	
	Solvent A (%)	Solvent B (%)		Solvent A (%)	Solvent B (%)
0	95	5	0	95	5
1	95	5	1	95	5
4	45	55	17	0	100
17	0	100	18	0	100
18	0	100	19	95	5
19	95	5	20	95	5
20	95	5			

Solvent A: water with 0.1% formic acid (by volume); Solvent B: acetonitrile with 0.1% formic acid (by volume)
