# Supplementary material for: Feces and liver tissue metabonomics studies on the regulatory effect of aspirin eugenol eater in hyperlipidemic rats
Source: Lipids Health Dis. 2017 Dec 11;16:240. doi: 10.1186/s12944-017-0633-0 (PMC5725792; doi:10.1186/s12944-017-0633-0)
Supplement: Supplementary file 3 — Effects of AEE on blood lipid levels in hyperlipidemic rats (n = 10). (PDF 44 kb) [file 12944_2017_633_MOESM3_ESM.pdf]

Additional file 3: Effects of AEE on blood lipid levels in hyperlipidemic rats (n = 10).

Variables	Control	Model	AEE
TG	0.44±0.15**	0.67±0.13	0.49±0.16**
HDL	0.52±0.05**	0.35±0.11	0.32 ±0.09$^{##}$
LDL	0.25±0.04**	0.46±0.07	0.31±0.09**
TCH	1.24±0.13**	1.63±0.16	1.22±0.2**

HFD: high fat diet; TG: triglyceride; HDL: high density lipoprotein; LDL: low density lipoprotein; TCH: total cholesterol. The unit of TG, TCH, HDL and LDL was mmol/L. $^{**}P < 0.01$ significant difference from model group; $^{##}P < 0.01$ significant difference from control group.
