# Supplementary material for: Feces and liver tissue metabonomics studies on the regulatory effect of aspirin eugenol eater in hyperlipidemic rats
Source: Lipids Health Dis. 2017 Dec 11;16:240. doi: 10.1186/s12944-017-0633-0 (PMC5725792; doi:10.1186/s12944-017-0633-0)
Supplement: Supplementary file 4 — Histopathological results of liver, stomach and duodenum after a five-week AEE treatment (HE × 100). (PDF 420 kb) [file 12944_2017_633_MOESM4_ESM.pdf]

Additional file 4: Histopathological results of liver, stomach and duodenum after a five-week AEE treatment (HE × 100).

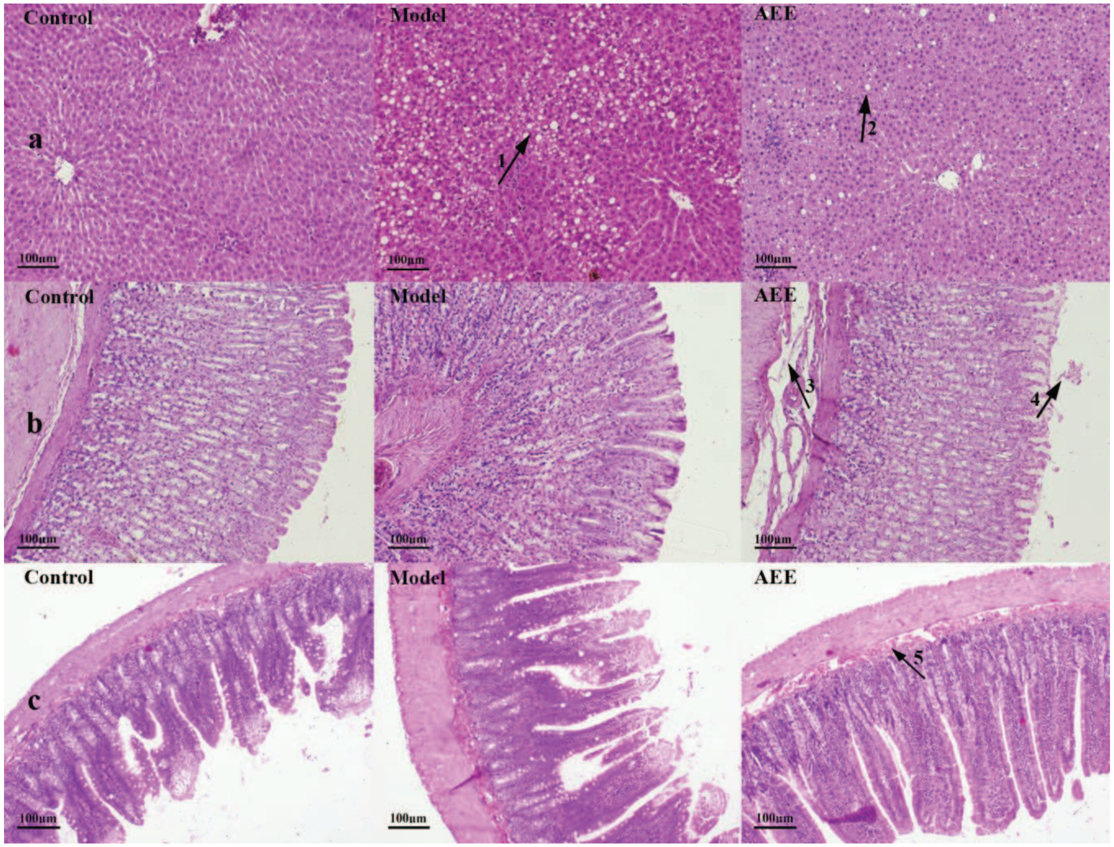

a: liver tissue; b: stomach; c: duodenum. In comparison with the model group, fatty degenerations and fat droplets of liver cells in AEE were significantly decreased (arrow 1-2). Minor pathological changes including hyperemia, edema and ecclasis were found (arrow 3-5).
