# Supplementary material for: Feces and liver tissue metabonomics studies on the regulatory effect of aspirin eugenol eater in hyperlipidemic rats
Source: Lipids Health Dis. 2017 Dec 11;16:240. doi: 10.1186/s12944-017-0633-0 (PMC5725792; doi:10.1186/s12944-017-0633-0)
Supplement: Supplementary file 6 — Fragments matched in HMDB or Metlin databases in metabolites identification in liver tissue. (PDF 49 kb) [file 12944_2017_633_MOESM6_ESM.pdf]

Additional file 6: Fragments matched in HMDB or Metlin databases in metabolites identification in liver tissue.

No.	Scan Model	Metabolite	Fragments MS/MS (Collision energy: 20 eV)
1	ESI+	Valine	55.0545; 56.0506; 57.0573; 72.0810
2	ESI+	Hypoxanthine	55.0292; 67.0292; 82.0401; 94.0399; 110.0346; 119.0349; 137.0450
3	ESI+	Phenylalanine	77.0387; 79.0542; 91.0541; 93.0696; 103.0541; 120.0805
4	ESI+	Glycocholic acid	76.0395; 227.1428; 319.2417; 337.2526; 412.2580; 430.2952
5	ESI+	LysoPC(18:0)	104.1068; 184.0726; 524.3713
6	ESI+	LysoPC(22:6)	86.0955; 104.1066; 184.0722; 550.3290; 568.3398
7	ESI+	LysoPC(20:4)	86.0966; 104.1069; 184.0722; 526.3297; 544.3409
8	ESI+	Palmitoylcarnitine	85.0284; 239.2352; 400.3424
9	ESI+	Stearoylcarnitine	267.2668; 369.3000; 428.3743
10	ESI+	Isoleucine	30.0331; 43.0542; 44.0497; 56.0498; 57.0570; 86.0967;
11	ESI+	Pantothenic acid	41.0383; 43.0176; 57.0698; 59.0492; 67.0541; 70.0288; 72.0445; 90.0547; 98.0235; 124.0754
12	ESI+	Tryptophan	91.0541; 115.0539; 117.0594; 118.0648;119.0678;132.0802; 142.0646; 143.0719; 144.0793; 146.0591
13	ESI+	Phytosphingosine	43.0543; 57.0701; 300.2889; 318.2998
14	ESI+	GUDCA	76.0392; 158.0806; 414.3001; 416.3065
15	ESI+	LysoPC(16:1)	86.0969; 104.1066; 184.0724; 494.3237
16	ESI+	Linoleyl carnitine	85.0283; 97.0994; 424.3420
17	ESI+	Glutamine	41.0386; 56.0494; 84.0443; 130.0497
18	ESI+	Oxidized glutathione	177.0314; 231.0418; 355.0735; 409.0838; 484.1161; 538.1266; 595.1478; 613.1586
19	ESI+	Niacinamide	51.0226; 52.0235; 53.0386; 78.0338; 80.0494; 96.0441; 123.0544
20	ESI+	TCDCA	126.0220; 208.0626; 464.2825; 466.2847
21	ESI+	LysoPC(20:5)	86.0963; 104.1068; 184.0725; 483.2459; 526.3293
22	ESI+	LysoPC(20:3)	86.0971; 104.1075; 184.0730; 528.3472; 546.3591

23	ESI-	Inosine	135.0308; 267.0724
24	ESI-	FAD	346.0547; 437.0854
25	ESI-	DHA	59.0143; 121.1017; 135.1172; 147.1176; 161.1327; 229.1956; 283.2423; 327.2318
26	ESI-	Arachidonic acid	59.0143; 83.0503; 205.1962; 231.2116; 259.2425; 285.2215; 303.2323
27	ESI-	Gluconic acid	57.0353; 59.0142; 71.0138; 75.0088; 87.0095; 129.0180
28	ESI-	Dephospho-CoA	408.0139; 686.1427
