# Supplementary material for: Feces and liver tissue metabonomics studies on the regulatory effect of aspirin eugenol eater in hyperlipidemic rats
Source: Lipids Health Dis. 2017 Dec 11;16:240. doi: 10.1186/s12944-017-0633-0 (PMC5725792; doi:10.1186/s12944-017-0633-0)
Supplement: Supplementary file 7 — Typical UPLC-Q-TOF/MS total ion chromatograms (TICs) of feces in positive and negative ion modes. (PDF 242 kb) [file 12944_2017_633_MOESM7_ESM.pdf]

Additional file 7: Typical UPLC-Q-TOF/MS total ion chromatograms (TICs) of feces in positive and negative ion modes

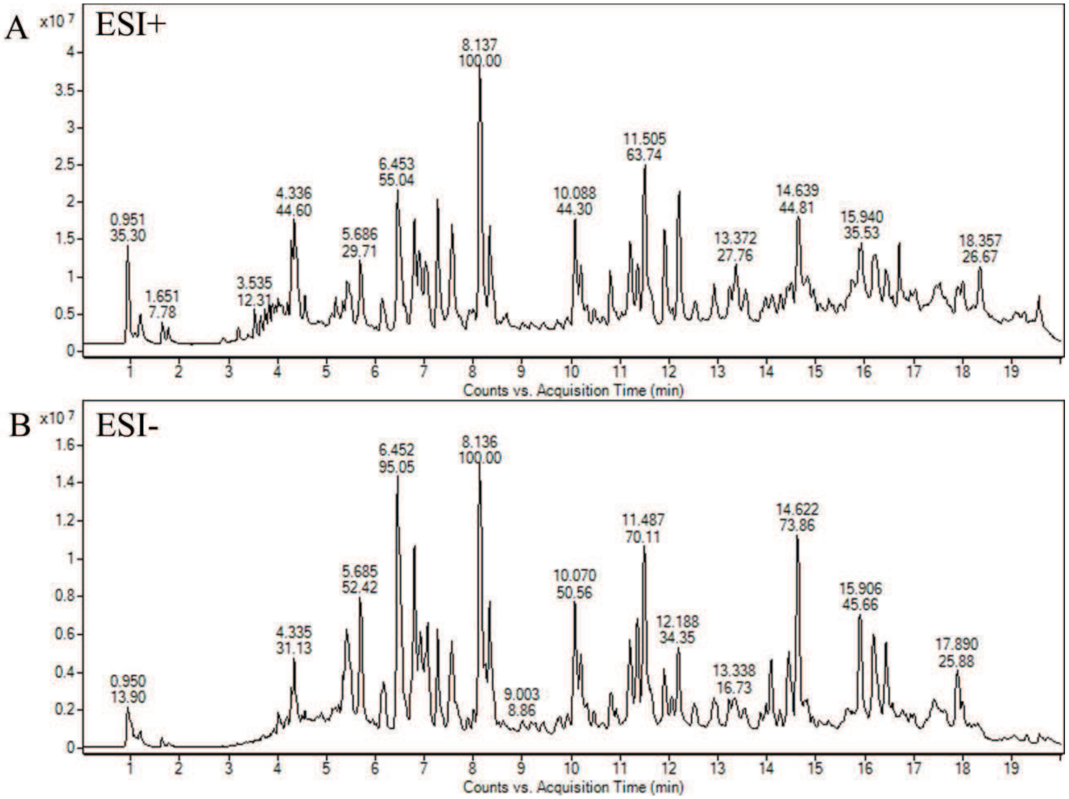

A: positive ion mode TIC; B: negative ion mode TIC.
