# Supplementary material for: Feces and liver tissue metabonomics studies on the regulatory effect of aspirin eugenol eater in hyperlipidemic rats
Source: Lipids Health Dis. 2017 Dec 11;16:240. doi: 10.1186/s12944-017-0633-0 (PMC5725792; doi:10.1186/s12944-017-0633-0)
Supplement: Supplementary file 8 — Fragments matched in HMDB or Metlin databases in metabolites identification in feces. (PDF 52 kb) [file 12944_2017_633_MOESM8_ESM.pdf]

Additional file 8: Fragments matched in HMDB or Metlin databases in metabolites identification in feces.

No.	Scan Mode	Metabolites	Fragments MS/MS (Collision energy: 20 eV)
1	ESI+	Nutriacholic acid	327.2674; 355.2630; 373.2732
2	ESI+	Phytosphingosine	57.0694; 60.0445; 282.2794; 300.2869; 318.3005
3	ESI+	Sphingosine	57.0699; 69.0699; 252.2678; 264.2690; 282.2783
4	ESI+	Linoleic acid	55.05449; 57.07008; 67.05437; 69.07001; 81.06986; 83.0855; 93.07004; 95.08559; 97.10097; 109.10105
5	ESI+	Stearoylcarnitine	85.0274; 144.1013; 267.2671; 428.3732
6	ESI+	Leucine	41.0385; 43.0543; 44.0496; 55.0543; 69.0698; 86.0963
7	ESI+	Tryptamine	91.0540; 115.0537; 117.0644; 127.0536; 143.0719; 144.0796
8	ESI+	Oleamide	43.0538; 55.0544; 57.0703; 67.0542; 69.0701; 79.0538; 81.0693; 282.2793
9	ESI+	Caprylic acid	29.0378; 39.0233; 41.0384; 43.0543; 53.0392; 55.0544; 73.0650
10	ESI+	PEA	55.0545; 57.0697; 67.0544; 69.0699; 81.0699; 83.0853; 95.0850; 97.1003; 93.0699; 280.2623
11	ESI+	Palmitic acid	43.0544; 55.0544; 57.0699; 67.0543; 69.0701; 71.0857; 81.0699; 83.0855; 95.0855
12	ESI+	Stearoylethanolamide	43.0544; 44.0497; 57.0699; 62.0602; 71.0855; 85.1009; 95.0851; 310.3098
13	ESI+	THA	81.0698; 95.0849; 109.0999; 121.1000; 135.1158; 149.1320; 161.1317; 215.1782; 357.2804
14	ESI-	4-(2-A)-2,4-DA	120.0451; 144.0456; 160.0400; 163.0556; 188.0343
15	ESI-	D-Phenyllactic acid	41.0045; 119.0498; 121.0654
16	ESI-	Enterolactone	107.0497; 119.0520; 121.0650; 189.0536; 253.1214
17	ESI-	13-HA	253.2533; 281.2463; 299.258
18	ESI-	Alpha-Linolenic acid	59.0137; 259.2035
19	ESI-	Oleic acid	263.2362; 281.2475
20	ESI-	Dodecanedioic acid	57.0350; 139.1128; 167.1435; 229.1437
21	ESI-	12-Ketodeoxycholic acid	343.2640; 389.2691
22	ESI-	DHA	59.0146; 121.1032; 229.1934; 283.2414; 327.2314

Additional file 9: Pathway analysis result with MetaboAnalyst 3.0

No.	Pathway Name	Total	Expected	Hits	Row *P*	-log (*P*)	Impact
1	Valine, leucine and isoleucine biosynthesis	11	0.24322	3	0.001434	6.5472	0.99999
2	Tryptophan metabolism	41	0.90656	3	0.059173	2.8273	0.19914
3	Phenylalanine, tyrosine and tryptophan biosynthesis	4	0.088445	1	0.085643	2.4576	0.5
4	Linoleic acid metabolism	5	0.11056	1	0.10592	2.2451	1
5	Phenylalanine metabolism	9	0.199	1	0.18276	1.6996	0.40741
6	alpha-Linolenic acid metabolism	9	0.199	1	0.18276	1.6996	1
7	Nicotinate and nicotinamide metabolism	13	0.28745	1	0.25318	1.3736	0.2381
8	Alanine, aspartate and glutamate metabolism	24	0.53067	1	0.41791	0.87249	0.14979
9	Arachidonic acid metabolism	36	0.79601	1	0.55748	0.58432	0.32601

Total: The total number of compounds in the pathways; the hits are the actually matched number from the user upload data; the raw p is the original p value calculated from the enrichment analysis; the impact is the pathway impact value calculated from pathway analysis.
