# Supplementary material for: Feces and liver tissue metabonomics studies on the regulatory effect of aspirin eugenol eater in hyperlipidemic rats
Source: Lipids Health Dis. 2017 Dec 11;16:240. doi: 10.1186/s12944-017-0633-0 (PMC5725792; doi:10.1186/s12944-017-0633-0)
Supplement: Supplementary file 9 — Pathway analysis result with MetaboAnalyst 3.0. (PDF 43 kb) [file 12944_2017_633_MOESM9_ESM.pdf]

Additional file 9: Pathway analysis result with MetaboAnalyst 3.0

No.	Pathway Name	Total	Expected	Hits	Row *P*	-log (*P*)	Impact
1	Valine, leucine and isoleucine biosynthesis	11	0.24322	3	0.001434	6.5472	0.99999
2	Tryptophan metabolism	41	0.90656	3	0.059173	2.8273	0.19914
3	Phenylalanine, tyrosine and tryptophan biosynthesis	4	0.088445	1	0.085643	2.4576	0.5
4	Linoleic acid metabolism	5	0.11056	1	0.10592	2.2451	1
5	Phenylalanine metabolism	9	0.199	1	0.18276	1.6996	0.40741
6	alpha-Linolenic acid metabolism	9	0.199	1	0.18276	1.6996	1
7	Nicotinate and nicotinamide metabolism	13	0.28745	1	0.25318	1.3736	0.2381
8	Alanine, aspartate and glutamate metabolism	24	0.53067	1	0.41791	0.87249	0.14979
9	Arachidonic acid metabolism	36	0.79601	1	0.55748	0.58432	0.32601

Total: The total number of compounds in the pathways; the hits are the actually matched number from the user upload data; the raw p is the original p value calculated from the enrichment analysis; the impact is the pathway impact value calculated from pathway analysis.
